# The radiomorphological appearance of the invasive margin in pancreatic cancer is associated with tumor budding

**DOI:** 10.1007/s00423-024-03355-3

**Published:** 2024-05-29

**Authors:** Philipp Mayer, Anne Hausen, Verena Steinle, Frank Bergmann, Hans-Ulrich Kauczor, Martin Loos, Wilfried Roth, Miriam Klauss, Matthias M Gaida

**Affiliations:** 1 Clinic for Diagnostic and Interventional Radiology, University Hospital Heidelberg, Heidelberg, 69120 Germany; 2 Institute of Pathology, University Medical Center Mainz, JGU-Mainz, Mainz, 55131 Germany; 3 Institute of Pathology, University Hospital Heidelberg, Heidelberg, 69120 Germany; 4 Clinical Pathology, Klinikum Darmstadt GmbH, Darmstadt, 64283 Germany; 5 Department of General, Visceral, and Transplantation Surgery, University Hospital Heidelberg, Heidelberg, 69120 Germany; 6 Translational Oncology, TRON, the University Medical Center, JGU-Mainz, Mainz, 55131 Germany; 7 Research Center for Immunotherapy, University Medical Center Mainz, JGU-Mainz, Mainz, 55131 Germany

**Keywords:** Pancreatic cancer, Magnetic resonance imaging, Tumor budding, Tumor cell invasion, Invasive margin

## Abstract

**Purpose:**

Pancreatic cancer (PDAC) is characterized by infiltrative, spiculated tumor growth into the surrounding non-neoplastic tissue. Clinically, its diagnosis is often established by magnetic resonance imaging (MRI). At the invasive margin, tumor buds can be detected by histology, an established marker associated with poor prognosis in different types of tumors.

**Methods:**

We analyzed PDAC by determining the degree of tumor spiculation on T2-weighted MRI using a 3-tier grading system. The grade of spiculation was correlated with the density of tumor buds quantified in histological sections of the respective surgical specimen according to the guidelines of the International Tumor Budding Consensus Conference (*n* = 28 patients).

**Results:**

64% of tumors revealed intermediate to high spiculation on MRI. In over 90% of cases, tumor buds were detected. We observed a significant positive rank correlation between the grade of radiological tumor spiculation and the histopathological number of tumor buds (r_s_ = 0.745, *p* < 0.001). The number of tumor buds was not significantly associated with tumor stage, presence of lymph node metastases, or histopathological grading (*p* ≥ 0.352).

**Conclusion:**

Our study identifies a readily available radiological marker for non-invasive estimation of tumor budding, as a correlate for infiltrative tumor growth. This finding could help to identify PDAC patients who might benefit from more extensive peripancreatic soft tissue resection during surgery or stratify patients for personalized therapy concepts.

**Supplementary Information:**

The online version contains supplementary material available at 10.1007/s00423-024-03355-3.

## Introduction

Pancreatic ductal adenocarcinoma (PDAC) is one of the most aggressive cancer entities, which is predicted to be the second cause of cancer-associated deaths in 2030 [1]. Since the majority of early-stage tumors display unspecific symptoms, usually, PDAC is detected in advanced stages, where to date only a minor group of patients qualify for potentially curative surgical resection [2]. PDAC is characterized by an infiltrating and unsharp, spike-like growth pattern [3]. Histomorphologically, PDAC shows mainly a glandular tumor growth pattern, however also other forms, such as (micro-) papillary, solid, cribriform, or single cellular dissociated tumor growth, respectively their combinations, can be seen [4]. At the invasive margin of pancreatic cancers, which are routinely evaluated in the surgical specimen, the neoplastic cells often form so-called tumor buds, which are either single cells or small clusters of up to four cells in dispersed arrangements and are thought to be associated with increased invasive behavior [5]. Tumor cells undergoing a budding process usually show de-differentiation and loss of polarity with an induced epithelial-to-mesenchymal transition as is seen in other epithelial cancers as well [6]. In colon cancer as well as in PDAC, tumor budding is described as a negative prognostic marker and is associated with lymph node and distant metastases [6, 7]. It is therefore suggested to mention the presence of tumor budding in the pathological report [7, 8]. Besides the classical EMT markers such as ZEB1, Snail, and Slug, also the factor N-Wasp was described as an important surrogate biomarker associated with tumor budding in colon carcinoma [9]. N-Wasp, which is a major protein responsible for cell invasion due to podosomes and invadopodia, is also expressed in PDAC and is associated with poor prognosis and metastatic behavior [10–12]. Although in contrast to colon cancer, tumor budding in PDAC does not yet have a direct impact on the choice of adjuvant therapy, non-invasive estimation of tumor budding might be helpful for improved prognostic stratification and prediction of outcome in PDAC patients [13, 14].

To the present, only a few studies have linked radiological imaging features with the presence and extent of tumor budding in various cancers. Chong et al. and Li et al. correlated radiological imaging features of cervical and rectal cancer with the tumor budding status using Radiomics [15, 16]. Radiomics is an advanced quantitative imaging analysis method that extracts features from radiological images that are difficult to appreciate with the naked eye [17]. However, Radiomics has not yet been widely integrated into routine radiological practice [17]. On the contrary, visually apparent radiological imaging features can be more easily integrated into the daily work of a radiologist and, in the case of PDAC, were reported to reflect well other histopathological and clinical parameters. On computed tomography (CT) and magnetic resonance imaging (MRI), most PDACs present as focal rather ill-defined lesions which are hypodense/ hypointense after intravenous contrast injection [18]. The dense fibroblastic stroma is held responsible for the typical slow contrast uptake of PDACs [18] and tumor enhancement patterns were shown to predict stromal content [19], identify biophysical PDAC subtypes [20], and are associated with patient outcome [21]. The ill-defined tumor margin can be attributed to the oftentimes dispersed histopathological growth pattern at the invasive front which means that tumor invasion in adjacent tissues occurs in the form of single cells or cell clusters rather than large cell aggregates [22]. Furthermore, changes at the tumor interface on radiological imaging can indicate response to chemotherapy [23]. Although CT remains the most widely used imaging modality for PDAC staging [18], MRI’s superior soft tissue contrast offers potential advantages for lesion detection and characterization [24].

In the present study, we investigated whether the appearance of the tumor interface on MRI corresponds to histopathological tumor budding at the invasive margin of PDAC as an acknowledged hallmark for diffuse and infiltrative tumor growth and poor prognosis.

## Materials and methods

### Patients

The study protocol was approved by the local Ethics Committee (S-044/2012). Informed consent was obtained from all individual participants included in the study. The study is a post-hoc analysis of prospectively acquired non-contrast MRI scans of the pancreas from two previous radiological studies [25, 26] in correlation with histopathological findings. The MRI scans were conducted on the day before surgery in patients who underwent resection of PDAC. Out of 51 available patients, 20 patients were excluded due to insufficient image quality in the anatomical imaging series. Evidence for any artifacts in individual slices (potentially hindering the assessment of subtle spiculae at the tumor interface) or motion between slices (potentially leading to missing depiction of parts of the tumor) in the transverse Half-Fourier Acquisition Single-Shot Turbo-Spin-Echo (HASTE) T2-weighted sequence in the pancreatic region led to the exclusion of the corresponding case. 3 patients were excluded since corresponding tissue slides for histological analysis were not available. The 28 remaining patients (12 women and 16 men; mean age 64.7 years ± standard deviation (SD) 11.3 years) were included in this study. From all patients, tissue of the operation specimen had been processed in the routine pathology and used for further analyses. A flowchart of the study population is shown in Supplementary Fig. 1. The detailed patient information is listed in Table 1.

**Table 1:** Clinical and pathological patient characteristics

Median Age (interquartile range)	64.5 years (56.5 to 72.0 years)
**Sex**	
Female Male	12 (42.9%) 16 (57.1%)
**T stage**	
T1 T2 T3 T4	0 (0.0%) 12 (42.9%) 16 (57.1%) 0 (0.0%)
**N stage**	
N0 N1 N2	5 (17.9%) 7 (25.0%) 16 (57.1%)
**M stage**	
M0/Mx M1	27 (96.4%) 1 (3.6%; distant abdominal lymph node metastases)
**Grading**	
G1 G2 G3	0 (0.0%) 18 (64.3%) 10 (35.7%)
**Tumor localization**	
Pancreatic head Pancreatic body and/ or tail	21 (75.0%) 7 (25.0%)
**Type of pancreatectomy**	
Whipple Left resection Total pancreatectomy	20 (71.4%) 6 (21.4%) 2 (7.1%)

### Magnetic resonance imaging

The pancreatic MRI protocol included at least HASTE T2-weighted imaging in transverse orientation, breath-hold T1-weighted in/opposed phase imaging in transverse orientation, and breath-hold diffusion-weighted (DW) magnetic resonance imaging in transverse orientation. The detailed MRI protocol is presented in Supplementary Table 1.

The radiological analyses were performed on the T2-HASTE images (repetition time (TR) 680 ms, echo time (TE) 95 ms, acquisition matrix 320 × 320, slice thickness 4 mm, gap 0.4 mm). Two radiologists with more than 10 and 3 years of experience in pancreatic imaging assessed the MRI scans in consensus, using a Picture Archiving and Communication System (PACS) workstation. The radiologists assessed the spiculation of the tumor interface using a three-point rating scale: Sp1: spiculation on less than one-third of the tumor perimeter; Sp2: spiculation on one to two-thirds of the tumor perimeter; Sp3: spiculation on more than two-thirds of the tumor perimeter.

### Scoring of tumor budding

All human tissue samples were diagnosed as ductal adenocarcinoma of the pancreas (PDAC), and provided by the local tissue bank in agreement with the regulations of the tissue bank and the approval of the local Ethics committee (no. 206/2005). All patients gave written informed consent. Tumor buds were analyzed by two independent pathologists (A.H., M.M.G) according to the official criteria released by the International Tumor Budding Consensus Conference (ITBCC) 2016 [7]. For this, hematoxylin and eosin (H&E) stained tissue sections and for confirmation, N-Wasp stained sections were analyzed at medium power (10x) on a light microscope (Olympus BX51; Olympus Germany, Hamburg, Germany) to identify the densest area of budding at the invasive margin of the tumor (hotspot area). Tumor buds were counted in the hotspot at 20x magnification and normalized to the specific microscope eyepiece field number diameter to determine the number of tumor buds per 0.785 mm^2^. Tumor buds were categorized in: Bd1: 0–4 buds/mm^2^, Bd2: 5–9 buds/mm^2^, Bd3: ≥10 buds/mm^2^.

### Immunohistochemistry

For confirmation and visualization of tumor buds, immunohistochemistry for N-Wasp was performed. In brief, the prepared paraffin sections of the respective tumor specimens (*n* = 28) were used for immunohistochemistry. After deparaffinization and re-hydration, heat-induced antigen retrieval was conducted using an acidic buffer (35 min; pH 6.0 Dako EnVision, Glostrup, Denmark), followed by overnight incubation with the primary antibody against N-Wasp. (1:100; Cell Signaling Technology, Leiden, Netherlands). After incubation with a secondary antibody (Dako), the binding was visualized with DAB + chromogen (Dako).

### Statistical analysis

Statistical data analysis was performed using MedCalc version 20.218 (MedCalc Software Ltd., Ostend, Belgium). Mann-Whitney U test was used for comparison of independent continuous or ordinal variables. Spearman rank correlation was used for correlation analyses. The significance level was set at 0.05.

## Results

### Spiculation of tumor interface on MRI

One-quarter of tumors (7 out of 28 cases; 25.0%) showed a high degree of spiculation on T2-HASTE images. 10 out of 28 tumors (35.7%) showed spiculation on less than one-third of the tumor perimeter (low spiculation) and 11 out of 28 tumors (39.3%) exhibited intermediate spiculation.

### Tumor budding

Analysis of (H&E) stained tissue sections using the established pathological consensus scoring system revealed tumor buds in 26 out of 28 cases (92.9%). The number of tumor buds ranged from 0 buds/ mm^2^ to 16.43/ mm^2^. The median number of tumor buds was 6.07/ mm^2^. For confirmation of tumor buds, N-WASP expression on tumor cells was detected in all tumors and facilitated analysis of buds.

The number of tumor buds did not differ between moderately differentiated (G2) and poorly differentiated tumors (G3, *p* = 0.456), between T2 tumors and T3 tumors (*p* = 0.352), or between node-negative and node-positive tumors (*p* = 0.588).

### Radio-pathological correlation

We observed a strong significant positive Spearman rank correlation between the degree of tumor spiculation on MRI and the histological density of tumor buds/mm^2^ (r_s_ = 0.745, *p* < 0.001). According to the Mann-Whitney U test, median tumor buds/mm^2^ were significantly higher in tumors with intermediate or high spiculation on MRI (*n* = 18, 10.71 buds/mm^2^, 95%CI 6.43 to 12.57 buds/mm^2^) compared to tumors with low spiculation (*n* = 10, 2.14 buds/mm^2^, 95%CI 0.34 to 3.23 buds/mm^2^, *p* < 0.001). Also, tumors with high spiculation on MRI (*n* = 7) had significantly more tumor buds/mm^2^ (median 12.14 buds/mm^2^, 95%CI 8.90 to 16.43 buds/mm^2^) compared to all other tumors (*n* = 21, median 2.86 buds/mm^2^, 95%CI 1.83 to 6.43 buds/mm^2^, *p* = 0.002). Vice versa, MRI spiculation scores were significantly higher in tumors with histological budding scores ≥ Bd2 (≥ 5 buds/mm^2^, *n* = 15, median spiculation score Sp2) than in tumors with budding score Bd1 (< 5 buds/mm^2^, *n* = 15, median spiculation score Sp1, *p* < 0.001).

**Fig. 1:**
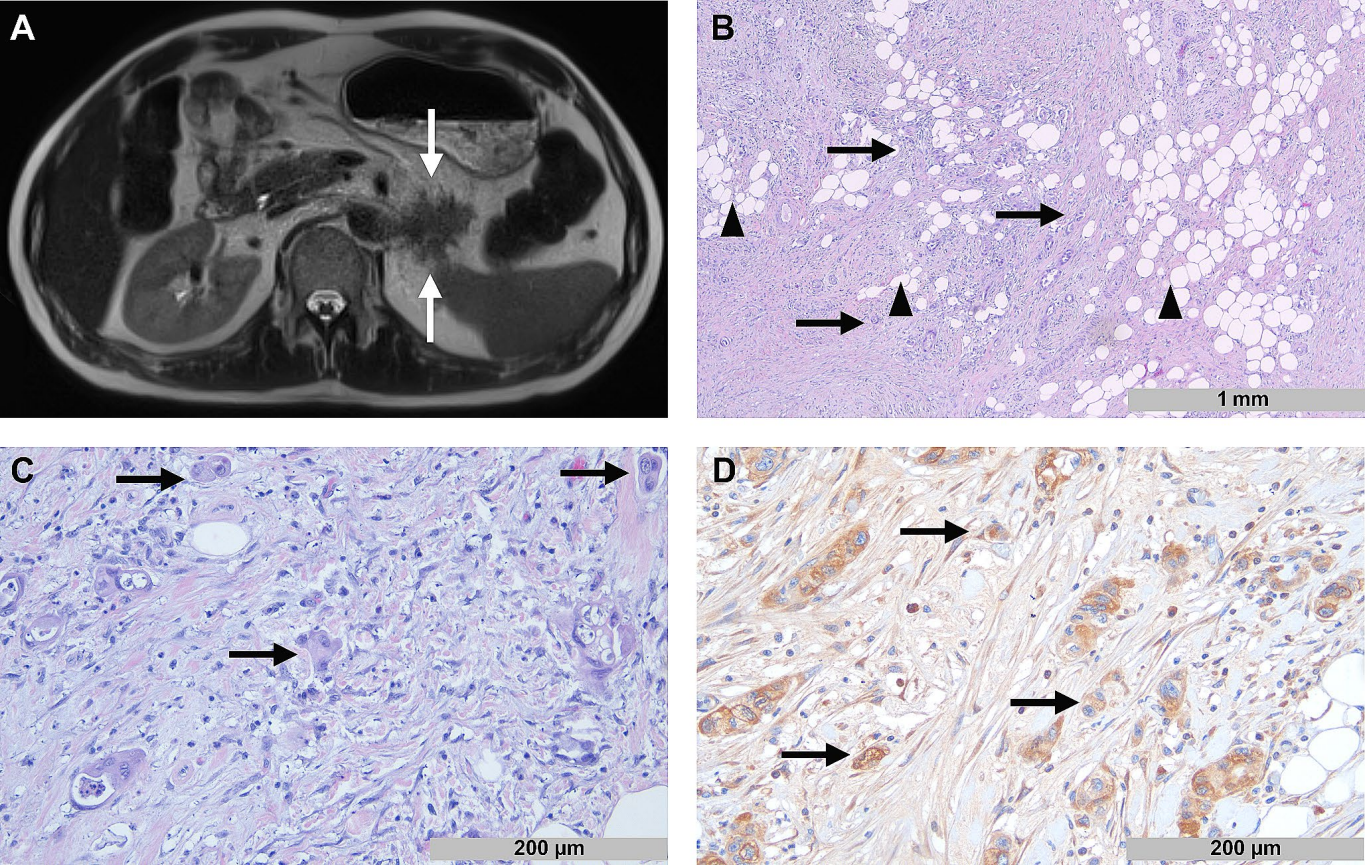
Radiopathological correlation in a patient with high tumor budding. **A**: Axial T2-HASTE image shows a pancreatic tail tumor with high spiculation (white arrows). **B**: Corresponding histopathological image shows diffuse infiltration of tumor cells (black arrows) into peripancreatic fat tissue (black arrowheads). **C**: Tumor cells form predominant clusters of up to four cells defined as tumor buds (black arrows). **D:** Tumor buds show expression of N-Wasp (black arrows)

**Fig. 2:**
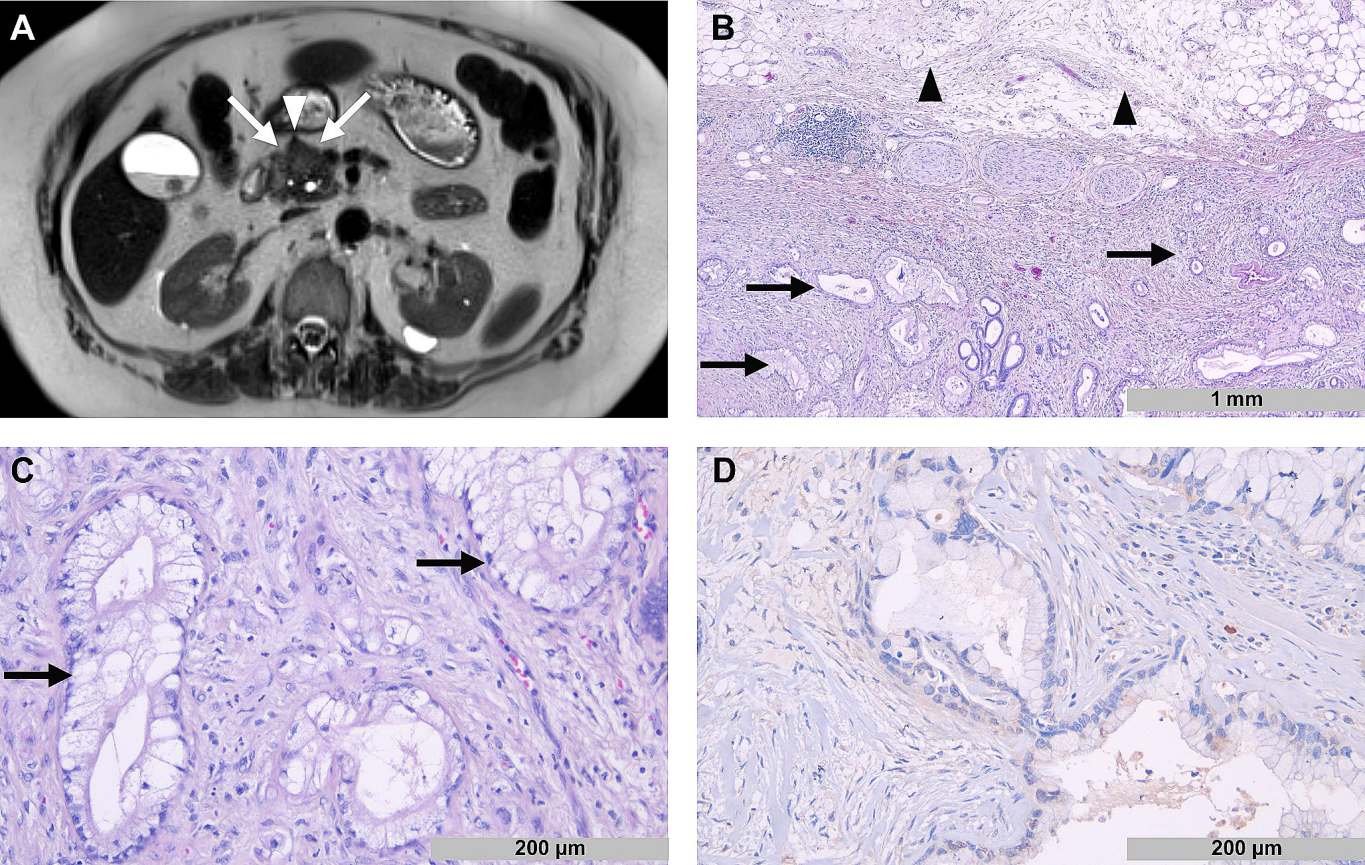
Radiopathological correlation in a patient with low tumor budding. **A**: Axial T2-HASTE image shows a tumor in the ventral part of the pancreatic head with low spiculation (white arrows). Note the small vessel at the tumor front (white arrowhead). **B**: In the corresponding histopathological image, tumor cells (black arrows) show a sharper margin towards peripancreatic fat tissue (black arrowheads). **C**: Tumor cells form predominantly ductular structures (black arrows). **D**: No significant expression of N-Wasp

Example images of a patient with high tumor budding and a patient with low tumor budding are shown in Figs. 1 and 2.

**Fig. 3:**
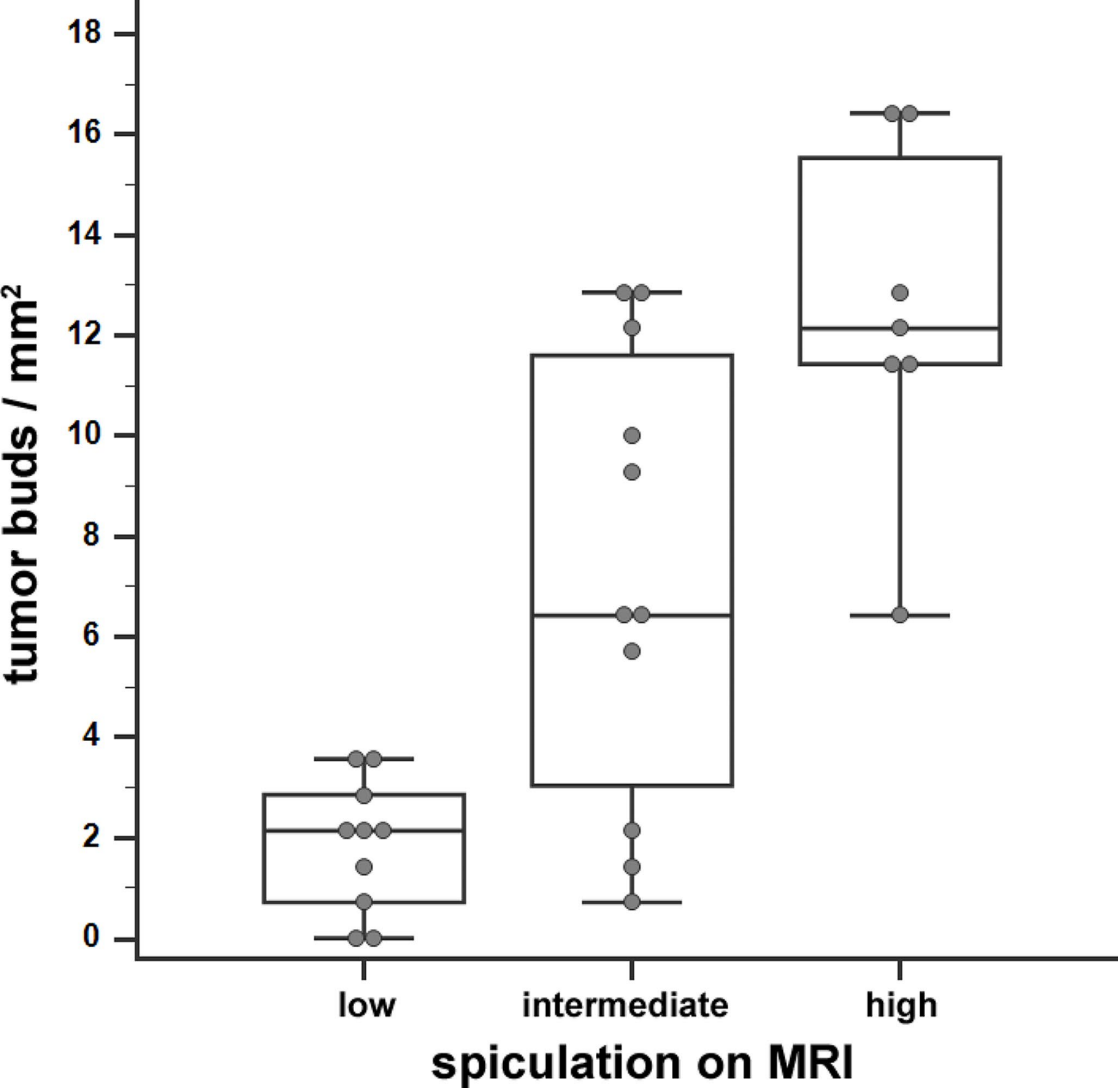
Box-and-whisker plot showing the distribution of histopathological tumor bud density relative to tumor spiculation on MRI. According to the Mann-Whitney U test, median tumor buds/mm^2^ were significantly higher in tumors with intermediate or high spiculation on MRI compared to tumors with low spiculation (*p* < 0.001). Tumors with high spiculation on MRI had significantly more tumor buds/mm^2^ compared to all other tumors (*p* = 0.002)

The distribution of histopathological tumor bud density relative to tumor spiculation on MRI is shown in Fig. 3.

**Fig. 4:**
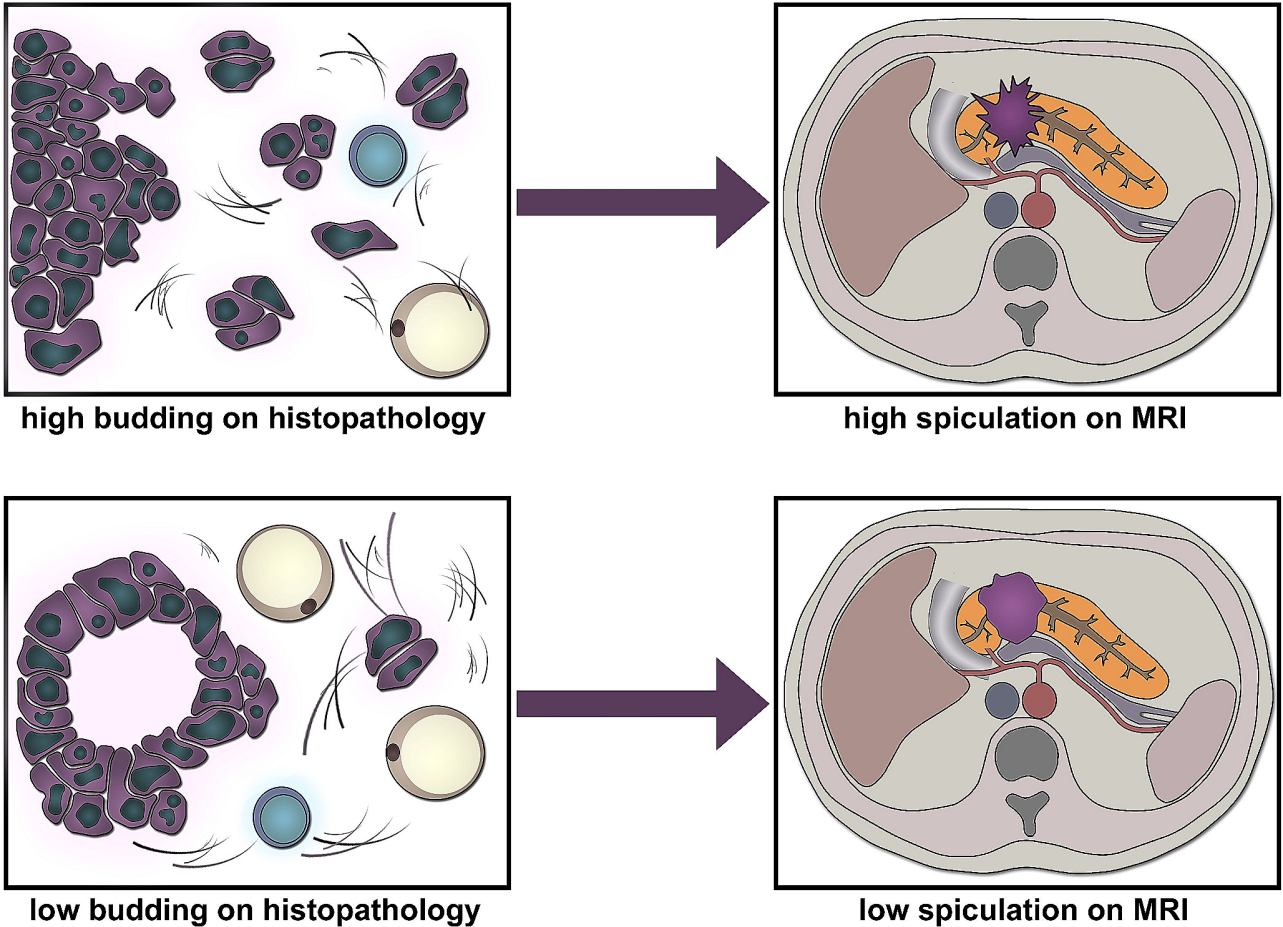
Graphical summary illustrating the association of histopathological tumor budding with the degree of tumor spiculation on MRI

A graphical summary illustrating the association of histopathological tumor budding with the degree of tumor spiculation on MRI is shown in Fig. 4.

The degree of spiculation on MRI did not differ between moderately differentiated (G2) and poorly differentiated tumors (G3, *p* = 0.319), between T2 tumors and T3 tumors (*p* = 0.181), or between node-negative and node-positive tumors (*p* = 0.774).

## Discussion

The present study shows that anatomical MR imaging of PDAC can predict the tumor budding status in PDAC, a tumor biological feature that is associated with aggressive and infiltrative growth behavior, metastatic spread, and poor outcome. Since tumor budding is a well-documented adverse risk factor for local recurrence, its preoperative assessment may help identify patients who could profit from more extended peripancreatic soft tissue removal during surgery [27]. On the other hand, tumor budding is also in the focus as part of personalized tumor therapies, especially for patients, who do not qualify for resection. Non-invasive tools for estimation may be beneficial for such therapeutic concepts. In this study, we observed a strong positive correlation between the grade of tumor spiculation on MRI and the histological density of tumor buds, the latter determined in histological slides using the established ITBCC consensus scoring system. Tumors with a high degree of spiculation on MRI showed a significantly higher density of buds compared to tumors with low to intermediate degrees of spiculation.

Studies on non-invasive prediction of tumor budding are scarce. A recent study by Chong et al. reported that MR-based radiomic features from T2-weighted imaging and contrast-enhanced T1-weighted imaging are associated with tumor budding status in cervical cancer patients treated with radical hysterectomy [15]. Li et al. in addition also integrated DW images into their multi-sequence radiomics model, which was able to predict the tumor budding status in locally advanced rectal cancer [16]. Although, being a well-accepted marker for tumor aggressiveness in PDAC [6, 7], its estimation using MRI has not yet been implemented in the clinical routine. This may be due to the high infrastructural needs of radiomics analysis or to a stronger focus on serum markers. A strength of this MRI-based study is its non-invasivity and in comparison to the approaches of Chong et al. and Li et al. [15, 16], the MR parameter analyzed in our study is less sophisticated and thus may be easily implemented into clinical practice.

In our study, about two-thirds of PDACs showed intermediate to high spiculation on MRI. This is in line with a previous MRI study on PDAC in which 80% of tumors appeared as ill-defined lesions [28]. This study found that well-defined tumor margins are rather atypical for PDAC and are more often observed in (non-hypervascular) neuroendocrine neoplasms [28]. Several CT studies reported similar rates of ill-defined tumor margins in PDAC [29–32] while one study reported that ill-defined margins are even more common in mass-forming pancreatitis than in PDAC [33]. Interestingly, in a study by Kim et al. from 2007, 93% of PDACs showed “well-defined demarcation” on MRI [34]. This discrepancy with the more consensual newer studies including the present study could be explained by the continuous improvements in MRI technology which enable a more detailed visualization of the tumor interface.

We detected tumor budding in the majority of tumors (92.9%) which is in accordance with studies by O’Connor et al. [35] and Chouat et al. [36]. It has been hypothesized that this high prevalence of tumor budding reflects PDAC’s aggressive biology and infiltrative growth [35] and various studies reported tumor budding to be a predictor of dismal prognosis in PDAC patients [36–39]. In colorectal cancer, where tumor budding has been most extensively investigated, it is strongly associated with higher T-stage and the presence of lymph node metastases [7]. This could either mean that the density of tumor buds increases in the course of disease progression and/ or that tumors with high numbers of tumor buds are more likely to progress to advanced stages. In PDAC, however, various studies, including the present, failed to prove significant association of tumor budding with T-stage or with lymphonodular metastases [36, 37, 40] while Karamitopoulou reported a significant association with higher T-stage [38] and Petrova et al. with N-stage [39]. The weaker association of tumor buds to pathological features may be due to additional factors, which may aggravate disease progression, such as early vascular invasion, extensive desmoplastic tumor stroma, or wide immune desertification [3].

In line with previous reports, no significant association of tumor budding with histopathological grading in the present study was seen, supporting the findings in the studies by Karamitopoulou et al. [38] and Chouat et al. [36], but being in contrast to other studies [35, 37, 39, 40]. Our data are supported by the release of the International Consensus Conference, which strictly separates histopathological grading from the amount of tumor budding, suggesting a localized effect in areas of the invasive tumor border [7]. These findings may be also related to the extraordinarily high tumor heterogeneity found in PDAC [3].

The study has limitations. First, the study cohort is rather small. This is the result of the rigorous exclusion of cases with any evidence of imaging artifacts or motion between slices in the pancreatic region in the T2-weighted HASTE sequence in order not to interfere with the assessment of slight details of the tumor interface. Patient anxiety and nervousness at the time of the MRI scan on the day before surgery might have also increased the prevalence of motion artifacts. The absence of cases with suboptimal imaging quality in the final study cohort could influence transferability into clinical practice. However, recent advances in (T2 HASTE) imaging, such as Deep Learning-accelerated imaging, can further reduce motion and blurring artifacts [41], thereby enabling the exact assessment of the tumor interface in a greater proportion of patients. Second, the present study is single-center. Future multi-center studies are desirable for the validation of our findings.

## Conclusion

Our study shows a widely applicable tool to estimate the density of tumor buds in PDAC using clinical MR imaging. The here proposed approach of non-invasive estimation of tumor budding by MRI could help to identify PDAC patients that might benefit from extended soft tissue resection during surgery since tumor budding is a strong indicator for incomplete (R1) resection [42]. Moreover, the non-invasive appraisal of tumor budding may as also be beneficial for prognostic stratification of PDAC patients [13] and – in the future with the possible advent of anti-budding therapies- stratification into therapeutic subgroups in times of personalized medical therapy concepts [14].

### Electronic supplementary material

Below is the link to the electronic supplementary material.

Supplementary Material 1

## Data Availability

The datasets generated during and/or analyzed during the current study are available from the corresponding authors upon reasonable request.
