# Supplementary material for: The radiomorphological appearance of the invasive margin in pancreatic cancer is associated with tumor budding
Source: Langenbecks Arch Surg. 2024 May 29;409(1):167. doi: 10.1007/s00423-024-03355-3 (PMC11136832; doi:10.1007/s00423-024-03355-3)
Supplement: Supplementary file 1 — Supplementary Material 1 [file 423_2024_3355_MOESM1_ESM.pdf]

Supplementary Material

The radiomorphological appearance of the invasive margin in pancreatic cancer is associated with tumor budding

Philipp Mayer[1*], Anne Hausen[2*], Verena Steinle[1], Frank Bergmann[3,4], Hans-Ulrich Kauczor[1], Martin Loos[5], Wilfried Roth[2], Miriam Klauss[1], Matthias M Gaida[2,6,7]

[1] Clinic for Diagnostic and Interventional Radiology, University Hospital Heidelberg, 69120 Heidelberg, Germany;

[2] Institute of Pathology, University Medical Center Mainz, JGU-Mainz, 55131 Mainz, Germany;

[3] Institute of Pathology, University Hospital Heidelberg, 69120 Heidelberg, Germany;

[4] Clinical Pathology, Klinikum Darmstadt GmbH, 64283 Darmstadt, Germany;

[5] Department of General, Visceral, and Transplantation Surgery, University Hospital Heidelberg, 69120 Heidelberg, Germany;

[6] TRON, Translational Oncology at the University Medical Center, JGU-Mainz, 55131 Mainz, Germany

[7] Research Center for Immunotherapy, University Medical Center Mainz, JGU-Mainz, 55131 Mainz, Germany

* Corresponding authors: Philipp Mayer, MD, PhD (Philipp.Mayer@med.uni-heidelberg.de) and Anne Hausen, MD (Anne.Hausen@unimedizin-mainz.de).

Supplementary Figure 1. Flowchart of the study population. The study is a post-hoc analysis of prospectively acquired non-contrast MRI scans of the pancreas from two previous radiological studies [25, 26].

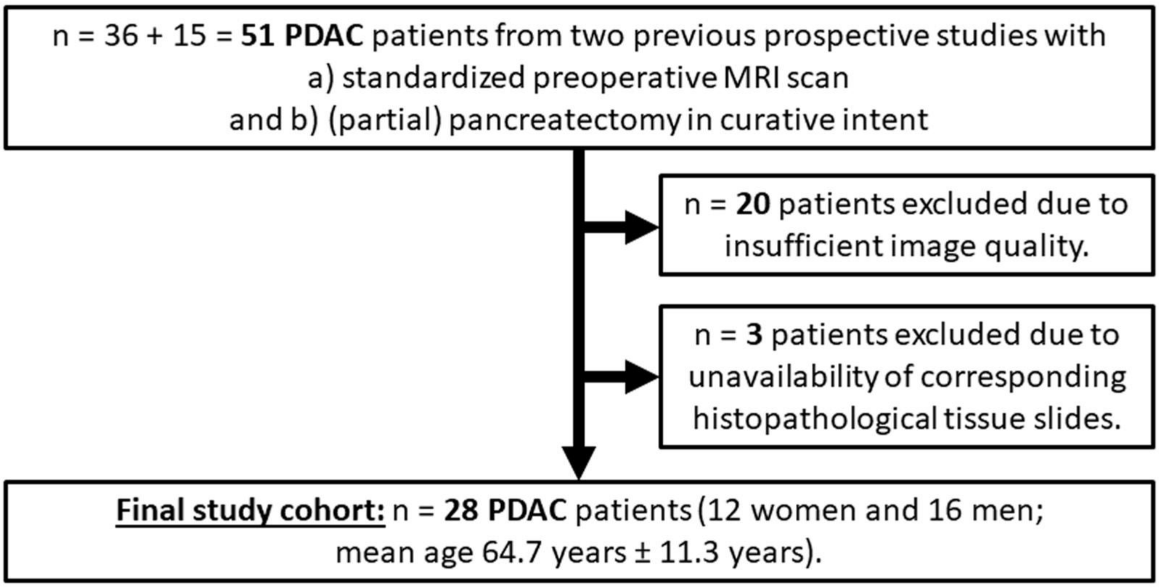

Supplementary Table 1. Acquisition parameters of the MRI protocol.

Sequence type	Acquired in	Orientation	Matrix of acquisition	Slice thickness/ gap	TR	TE [ms]	b-values
HASTE T2-weighted	breath-hold (expiratory)	Transversal	320 x 320	4 mm/ 0.4 mm	680 ms	95 ms	
HASTE-IR T2-weighted	breath-hold (inspiratory)	Coronal	256 x 230	6 mm/ 0.6 mm	1000 ms	80 ms	
T1-weighted in/opposed phase	breath-hold (inspiratory)	Transversal	320 x 272	5 mm/ 1 mm	115 ms	2.27 ms & 4.78 ms	
Diffusion-weighted imaging: ss-EPI	Breath-hold (expiratory)	Transversal	130 x 92	5 mm / 0.25 mm	2200 ms	58 ms	0, 50, 100, 150, 200, 300, 400, 600, and 800 s/mm^2

Abbreviations: HASTE, half-Fourier acquisition single-shot turbo spin-echo; IR, inversion recovery; ss-EPI, single-shot echo-planar imaging; TE, echo time; TR, repetition time.
